# In Vivo Contrast Imaging of Rat Heart with Carbon Dioxide Foam

**DOI:** 10.3390/s22145124

**Published:** 2022-07-08

**Authors:** Anton Karalko, Peter Keša, Frantisek Jelínek, Luděk Šefc, Jan Ježek, Pavel Zemánek, Tomáš Grus

**Affiliations:** 1 University General Hospital, First Faculty of Medicine, Charles University, U Nemocnice 499/2, 128 08 Prague, Czech Republic; anton.karalko@gmail.com (A.K.); tomas.grus@vfn.cz (T.G.); 2 Klinikum Neumarkt, Vascular Surgery, Nürnberger Str. 12, 92318 Neumarkt, Germany; 3 Center for Advanced Preclinical Imaging (CAPI), First Faculty of Medicine, Charles University, Salmovska 3, 120 00 Prague, Czech Republic; sefc@cesnet.cz; 4 Veterinary Histopathological Laboratory, Sojovicka 352/16, 197 00 Prague, Czech Republic; jelinekvet@seznam.cz; 5 Institute of Scientific Instruments, Czech Academy of Sciences, Kralovopolska 147, 612 64 Brno, Czech Republic; jezek@isibrno.cz (J.J.); pavlik@isibrno.cz (P.Z.)

**Keywords:** carbon dioxide foam, angiography, contrast-enhanced ultrasound imaging, preclinical imaging

## Abstract

Widely used classical angiography with the use of iodine contrast agents is highly problematic, particularly in patients with diabetes mellitus, cardiac and pulmonary diseases, or degree III or IV renal insufficiency. Some patients may be susceptible to allergic reaction to the iodine contrast substance. The intravenous injection of a bolus of CO_2_ (negative contrast) is an alternative method, which is, however, currently only used for imaging blood vessels of the lower limbs. The aim of our project was to design and test on an animal model a methodology for injecting the CO_2_ foam which would minimize the possibility of embolization of the brain tissue and heart infarction, leading to their damage. This is important research for the further promotion of the use of CO_2_, which is increasingly important for endovascular diagnosis and treatment, because carbon-dioxide-related complications are extremely rare. CO_2_ foam was prepared by the rapid mixing in a 2:1 ratio of CO_2_ and fetal bovine serum (FBS)-enriched Dulbecco’s Modified Eagle Medium (DMEM). Freshly prepared CO_2_ foam was administered into the catheterized rat tail vein or cannulated rat abdominal aorta and inferior vena cava (IVC). CO_2_ foam was compared with commercially available microbubbles (lipid shell/gas core). The rat heart in its parasternal long axis was imaged in B-Mode and Non-linear Contrast Mode before/during and after the contrast administration. Samples of the brain, heart and lungs were collected and subjected to histological examination. The non-linear contrast imaging method enables the imaging of micron-sized gas microbubbles inside a rat heart. The significantly shorter lifetime of the prepared CO_2_ foam is a benefit for avoiding the local ischemia of tissues.

## 1. Introduction

Angiography based on an iodine contrast agent is the gold standard in clinical diagnostics. Iodinated contrast agents are routinely used in procedures to diagnose and treat peripheral vascular disease. Despite the development of low-osmolar contrast agents and premedication techniques, these agents are still associated with contrast-induced nephropathy, allergic reactions or brain embolization in some individuals. To overcome these problems, carbon dioxide angiography has been developed as an alternative to standard iodinated contrast angiography in certain patient populations. Dioxide angiography has become a widely used modality for vascular imaging and endovascular procedures [1,2]. Its applications include digital subtraction angiography, aortography and venography, imaging for thrombosed AV-fistula [3,4,5,6]. Pure CO_2_ gas is used in peripheral arterial interventions and endovascular procedures of aneurysmal pathologies of the infrarenal aorta and its branches, detection of endoleaks after stent graft implantation and as an alternative approach to the routinely used iodine contrast in angiography by ultrasound imaging [7]. CO_2_ is used instead of air because it rapidly dissolves in the blood and only forms a temporary vein occlusion. Another technique, contrast-enhanced ultrasound (CEUS) imaging has found increasing use in preclinical and clinical imaging, because it does not involve radiation and has no harmful effects on diagnosed tissues [8]. CEUS is able to detect and assess microbubbles down to 2–3 µm, which is below the detection limit of Doppler techniques [9]. CEUS uses micron-size microbubbles that scatter non-linear ultrasound waves as a contrast agent in angiography in preclinical and clinical imaging [10].

Gas-filled microbubbles can be fabricated by the mixing of gas with an isotonic solution, or a commercial microbubble contrast agent made from reconstituted lyophilized lipid-shell gas-core contrast agents can be used [11]. Encapsulated CO_2_ microbubbles were employed as a contrast agent for the preclinical imaging of portal vein embolization [12].

The aim of this work is the in vitro and in vivo preclinical evaluation of the CO_2_ microbubble contrast agent in the form of foam compared to the commercially available microbubble contrast agent Vevo MicroMarker^®^ (Bracco, Geneva, Switzerland). Both of the above-mentioned contrast agents were used for the real-time in vivo imaging of rat heart. We evaluated the CO_2_ foam as a contrast agent and determined whether its rapid dissolution prevents ischemic tissue changes. Our approach is also cheap and time-saving. Understanding the unique properties of CO_2_ foam, the techniques for its use and its associated limitations and complications will allow interventional specialists to expand their options for the diagnosis and treatment of atherosclerotic peripheral vascular disease.

## 2. Materials and Methods

### 2.1. Animal Handling

We used 12 adult male Lewis rats (LEW/Crl, 12–14 weeks old, 270–300 g weight) in this study. The rats were purchased from Charles River animal facility (Sulzfeld, Germany) and bred in house. They were maintained in individually ventilated cages (Tecniplast, Buguggiate, Italy) under stable breeding conditions (12/12 h light/dark cycle, 22 ± 1 °C, 60 ± 5% humidity). The animals were supplied with a standard breeding diet for mice and rats (Altromin, Spezialfutter GmbH & Co. KG, Lage, Germany) and water *ad libitum*. All surgical procedures and in vivo imaging were approved by the Governmental Animal Care and Use Committee (MSMT-46307/2020-3).

### 2.2. Carbon Dioxide Foam Preparation

CO_2_ foam was made using a three-way stopcock as follows: two 6 mL three-part syringes (Terumo, Tokyo, Japan) were connected to the valve. The first was filled with 2 mL of high-glucose Dulbecco’s Modified Eagle’s Medium (DMEM medium) enriched with 10% fetal bovine serum (both Sigma Aldrich, St. Louis, MO, USA), while the second was filled with 4 mL of pure CO_2_ gas directly from a CO_2_ pressure cylinder. The volume of both syringes was then mixed 50 times as fast as possible. Freshly prepared foam was immediately administered into a cannulated animal.

### 2.3. In Vitro Optimization of Foam Production

The CO_2_ foam was prepared by mixing DMEM&FBS (10% FBS) and CO_2_ in ratios of 1:1, 1:2, 1:3 and 1:5. Freshly prepared foam was tested as follows: 1 mm (inner diameter) tubing was filled with foam, inserted into the plastic holder, and immersed in water at ambient temperature. Then, the NLC signal was acquired for 10 min for each DMEM&FBS:CO_2_ ratio.

### 2.4. Surgery Procedures

Rats were initially anesthetized by the spontaneous inhalation of isoflurane (3%—1.2 L/min air flow) inside a plastic cage using an isoflurane vaporizer (FUJIFILM Inc.). After that, animals were maintained under anesthesia by the inhalation of 2% isoflurane using a nose cone and fixed onto a heated table (39 °C). The site of the intervention was shaved using hair remover shaving gel (Strep, Milan, Italy) and disinfected with 70% ethanol. An approximately 2.5 cm skin incision was made above the area of interest. In front of and behind the entrance for the polyethylene cannula (BTPE-10, 0.28 × 0.60 mm) (Instech Laboratories Inc., Plymouth Meeting, PA, USA), the vein (infrarenal aorta, inferior cava vena) was tied up with two 8/0 Ethilon^TM^ sutures (Ethicon, LLC, Blue Ash, OH, USA) to avoid massive blood loss. Then, the vein (and/or artery) was punctured with a 23 G needle (Braun, Melsungen, Germany) and the moistened cannula (0.28 mm outer diameter) with an inserted guide wire (“0.010” diameter, ASAHI RG3, Asahi Inyecc Hanoi CO., LTD, Hanoi, Vietnam) was carefully introduced into the vein and sutured using a 10/0 Ethilon^TM^ suture (Ethicon, LLC). After successful animal cannulation, the skin closure was performed with a 5/0 Ethilon^TM^ suture (Ethicon, LLC).

### 2.5. Non-Linear Contrast (NLC) Imaging

Ultrasound and non-linear contrast imaging of rats was carried out using a multimodal Vevo 3100 high-frequency ultrasound imaging platform (FUJIFILM VisualSonics, Toronto, ON, Canada). An Mx201 ultrasound transducer (110 µm axial and 150 µm lateral resolution, 256 elements linear array, FUJIFILM VisualSonics) operating at a working frequency of 12.5 MHz (NLC-Mode) was used for all records. The rats were placed onto a heated table (FUJIFILM VisualSonics) to enable the monitoring of vital functions (ECG and breathing). The contrast agent (2 mL of CO_2_ foam or 220 µL of commercial microbubble contrast agent made of 110 µL Vevo MicroMarker microbubbles mixed with 110 µL of saline) was administered via 24-gauge cannula. The 2 mL of CO_2_ foam was administered within 5 s. The rat’s heart was visualized in parasternal long axis (PSLAX) view immediately after surgery using an Mx201 transducer by hand. The microbubbles were monitored in the rat’s heart (PSLAX) in B-Mode and Non-linear Contrast Mode with settings for all scans adjusted as follows: B-Mode Gain: 22 dB, Dynamic Range: 35 dB, Contrast Gain: 30 dB, Power: 4%, Gate: 5, Beamwidth: wide. The total image depth in the z-axis (field of view) was 26 mm, and the gap between the animal’s skin and the transducer surface (3 mm) was filled with a transparent ultrasound gel.

### 2.6. Hematoxylin/Eosin Staining of Tissues

Samples of organs were fixed in 10% neutral buffered formalin and processed by the common paraffin method. Histological sections 5 µm thick were stained with hematoxylin and eosin.

### 2.7. Data Postprocessing and Statistical Analysis

The data obtained from in vitro and in vivo measurements were analyzed in detail with the software VevoLAB V. 3.2.5. and Vevo CQ (FUJIFILM VisualSonics). The parameters Peak Enhancement (a.u.) and Wash-in Rate (a.u.) were assessed, which characterize the microbubble size and foam absorption in the blood, respectively. For in vivo records, the speed of contrast agent inflow into the right atrium, left atrium, aorta and left ventricle was assessed by drawing the proper area over each chamber and aorta separately.

The statistical analysis of obtained numerical data was performed using the software Origin Pro 8 (OriginLAB Corporation, Northampton, MA, USA). A one-way ANOVA test was used, and the means were compared with each other (Tukey’s test). Results with *p*-values < 0.05 were assessed as statistically significant.

## 3. Results

### 3.1. In Vitro Stability of Carbon Dioxide Foam

**Figure 1:**
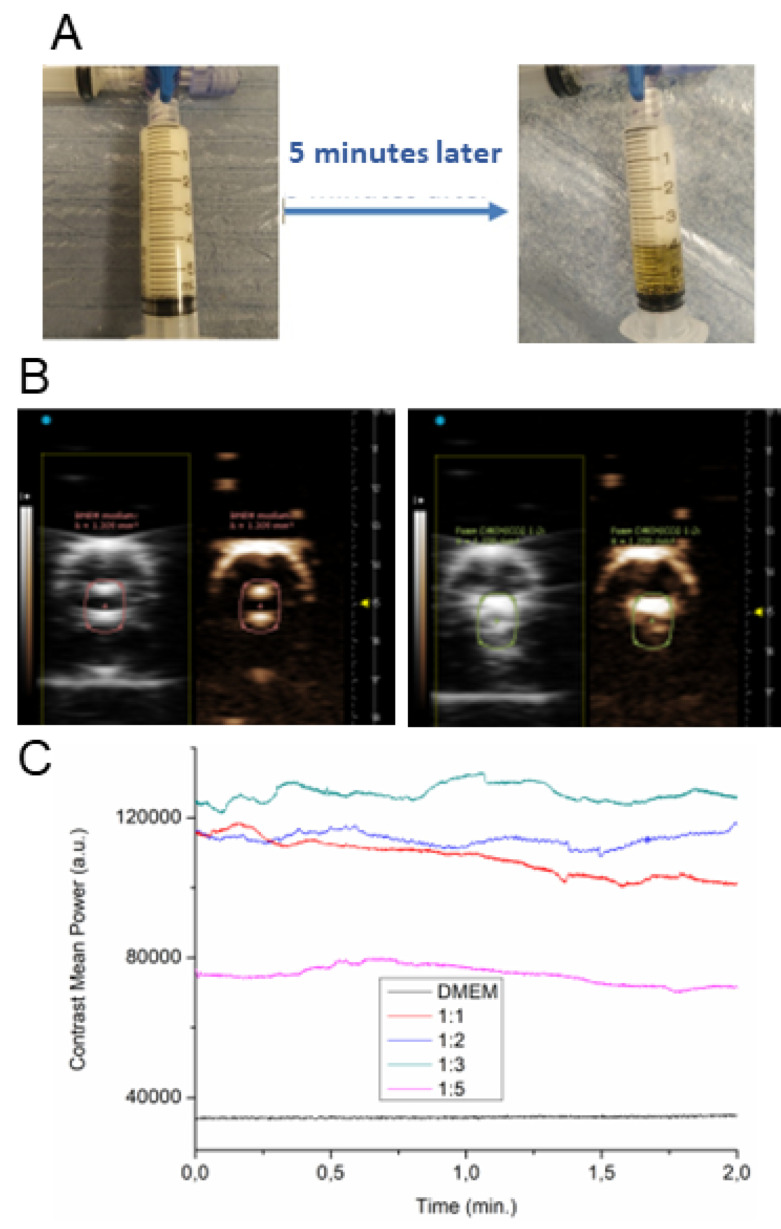
In vitro evaluation of CO_2_ foam. (**A**) CO_2_ foam prepared by mixing DMEM&FBS medium and carbon dioxide (1:2) immediately after preparation (left picture) and 5 min later (right). (**B**) NLC imaging of CO_2_ foam in phantoms. US (left) and NLC phantom imaging (right) of DMEM&FBS medium (left panel) and DMEM&FBS:CO_2_ 1:2 *v*/*v* foam (right panel). (**C**) Contrast signal obtained from pure DMEM&FBS medium and different DMEM&FBS:CO_2_ ratios.

The stability of freshly prepared CO_2_ foam was assessed directly in the syringe as the ratio of the foam volume to liquid volume over time, and by phantom measurements using silicone tubing. The CO_2_ foam (1:2) was stable for approximately five minutes. After 5 min, encapsulated CO_2_ gas was slowly lost (Figure 1A). Keep in mind the static conditions. In vitro phantom measurements were performed to find the highest NLC signal (see Figure 1B) of foam prepared by mixing of DMEM&FBS and CO_2_ in different ratios. The ratio 1:2 achieved the highest NLC signal stability during the acquisition (see Figure 1C) compared to pure DMEM&FBS. The intensity of the contrast signal (contrast mean power is a function of time in a region of interest) is proportional to the ultrasound contrast agent concentration. This is the way to achieve qualitative control of the prepared foam.

The CO2&DMEM ratios 2:1 and 3:1 gave the best foam stability (see Figure 1C). We decided to use the 2:1 gas mixture for in vivo experiments.

### 3.2. In Vivo NLC Imaging of Rat Heart

Contrast-enhanced ultrasound enables the non-invasive imaging and assessment of freshly prepared CO_2_ foam and Vevo MicroMarker^TM^. First, the signals of both CO_2_ foam and Vevo MicroMarker^TM^ microbubbles after their administration to the rat tail vein were compared. Second, both freshly prepared contrast agents were used for in vivo imaging of the rat heart in PSLAX view after administration of the contrast agent into the cannulated infrarenal aorta and inferior vena cava.

**Figure 2:**
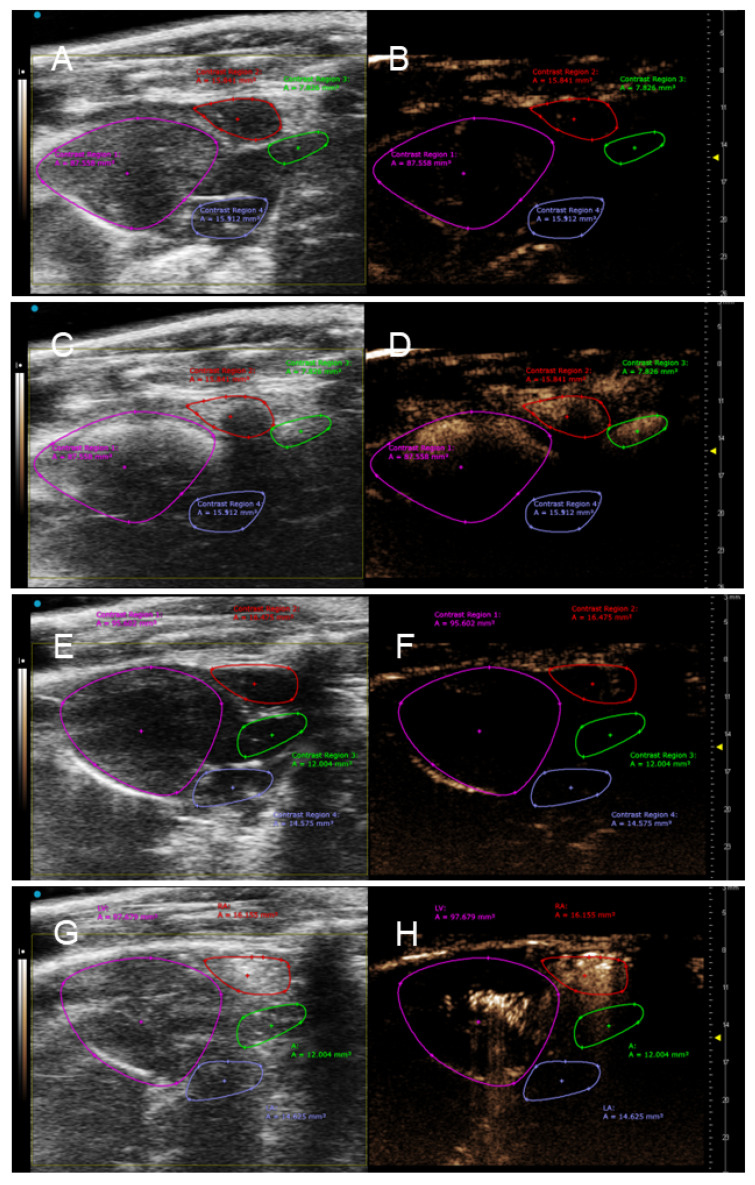
In vivo imaging of rat heart after administration of two different contrast agents. Representative images of rat heart in PSLAX before intravenous tail vein administration of 220 µL Vevo MicroMarker (**B**) and 2 mL of CO_2_ foam (**F**) and 5 s later (**D**,**H**). Corresponding images in B-Mode are in the left panel (**A**,**C**,**E**,**G**). The NLC signal was observed inside the right atrium (red), left atrium (blue), aorta (green) and left ventricle (purple).

The intravenous application of 2 mL of CO_2_ foam (*n* = 3) and 220 µL of Vevo MicroMarker^TM^ microbubble contrast (*n* = 3) as contrast agents into the rat tail vein led to an increase in the NLC signal in the right atrium, left ventricle and the aorta immediately after the contrast agent’s administration (see Figure 2) in all six experimental rats.

Important parameters such as Peak Enhancement (PE) and Wash-in Rate (WiR) represent bolus kinetics (PE relates to blood volume and WiR the maximum slope of the Peak Enhancement). Thus, lower WiR indicates poor perfusion and vice versa. Data obtained from these measurements are in Table 1. Both PE and WiR were higher (2480 ± 1030 a.u. and 3400 ± 2684 a.u.) in the right atrium after foam administration than with Vevo MicroMarker™ (636 ± 141 a.u. and 447 ± 153 a.u.). On the other hand, higher values of both parameters PE and WiR of the Vevo MicroMarker™ microbubbles (929 ± 264 and 2645 ± 2037) than for CO_2_ foam (363 ± 230 and 1688 ± 1596) indicate their prolonged circulation in the blood.

**Figure 3:**
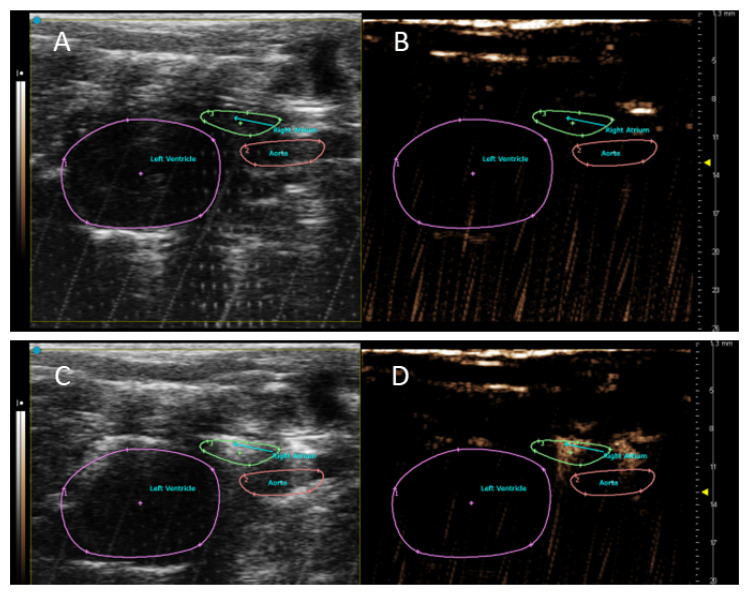
NLC imaging of rat heart with CO_2_ foam in PSLAX view. The experimental rat was cannulated in the inferior vena cava (IVC). The heart before (**B**) and 5 s after administration (**D**) of 2 mL of CO_2_ foam. Corresponding images in B-Mode are in the left panel (**A**,**C**). The NLC signal was observed inside the right atrium (green) and pulmonary artery.

The signal originating from the CO_2_ foam was detectable in the heart chambers after its administration into the cannulated inferior vena cava (see Figure 3).

**Figure 4:**
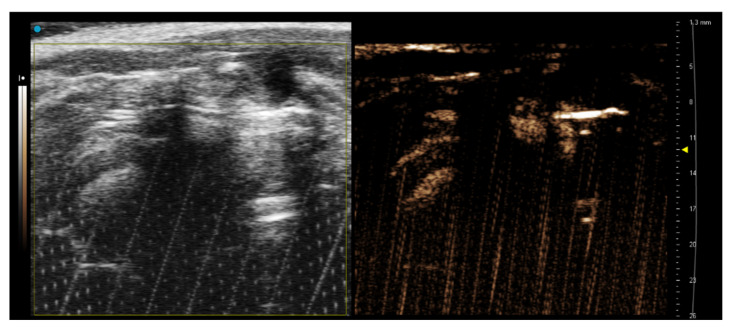
NLC imaging of rat heart 5 s after administration of Vevo MicroMarker contrast agent in PSLAX view. The rat was cannulated in the infrarenal aorta. A successful administration of Vevo MicroMarker contrast agent in B-Mode (**left**) and NLC Mode (**right**) was detectable in the RA and LV and aorta.

The cannulated infrarenal aorta (Figure 4) was only a useful approach for the administration of Vevo MicroMarker™. The administration of 2 mL of CO_2_ foam into the infrarenal aorta did not affect the NLC signal in the heart chambers.

### 3.3. Hematoxylin/Eosin Staining–Histological Examination

To investigate the potential side effects of CO_2_ foam on the brain, myocardium and lung ischemia that can occur after CO_2_ administration, tissues were subjected to hematoxylin eosin histological analysis. The samples were collected 24 h after CO_2_ foam administration.

**Figure 5:**
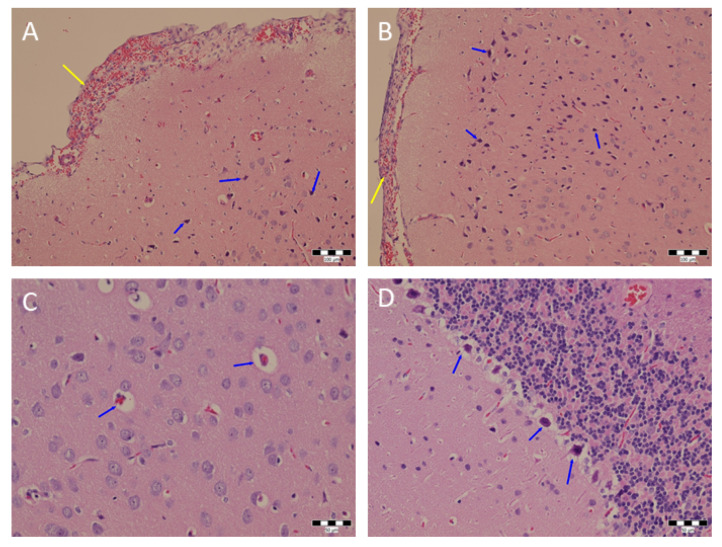
Histological analysis of brain tissue stained with hematoxylin and eosin. Hemorrhage in the meninges of the hemisphere (yellow arrow). Some of the dark neurons in the cortex are indicated with blue arrows (**A**). Hyperemia and hemorrhage in the meninges of the cerebral hemisphere (yellow arrow). Some of the dark neurons in the cortex are indicated with blue arrows (**B**). Pericapillary edema in the cortex of the hemisphere (blue arrows) (**C**). Dark neurons in the Purkinje cell zone in the cerebellum. Some of them are marked with blue arrows (**D**). Scale bar 100 µm.

The hematoxylin eosin staining did not confirm any significant damage to the brain, heart or lung tissues. The sample of the first rat brain (Figure 5) had dark neurons that were situated in the vicinity of small pyramidal neurons and the inner layer of granular neurons and in the hypothalamus. Dark neurons were also found in the hippocampus (second and third rats), in the thalamus and hypothalamus (third rat only), Purkinje zone of the cerebellum and in the medulla oblongata. The control rat brain had a smaller number of dark neurons in the cortex of the hemispheres, in the hippocampus and in the midbrain. The heart of the first rat had no pathological changes. Heart chamber dilatation was found in the sample of the second rat. Acute venostasis without further pathological lesions was found in the heart sample of the third rat. Mild acute venostasis was detected 24 h after CO_2_ foam administration in the control heart. The infiltration of the interstitium of lung tissue by histiocytes with both large and fine vacuoles in the cytoplasm, mild hyperemia and mild vicarious emphysema was found in the sample from the first animal. Mild to moderate hyperemia and infiltration of a part of the pulmonary interstitium by histiocytes was observed in the second rat. Mild acute venostasis with hemorrhagic foci was found in the lung sample of the third animal accompanied by occasional mild infiltration of the interstitium by histiocytes and mild acute emphysema. In contrast, the control lungs exhibited acute venostasis and a large area of infiltration of the pulmonary interstitium by histiocytes with finely vacuolated cytoplasm and vicarious emphysema.

## 4. Discussion

Carbon dioxide as a bolus injection of gas replacing blood in vessels has been used as a contrast agent for arteriography since the 1970s [13]. It is an odorless gas that has a negative contrast on CT; it is compressible and has a lower viscosity than iodine contrast agents. For safety reasons, the use of carbon dioxide bolus as a contrast agent is recommended for imaging vessels below the level of the diaphragm. The accuracy of angiography with CO_2_ is reportedly slightly lower; however, the risk of contrast nephropathy is excluded, regardless of the volume. Given the buoyancy of CO_2_, it may be less reliable at filling peripheral parts of the vascular bed in a dependent position and may cause obstruction due to the “vapor lock phenomenon” [14].

Carbon dioxide can be mixed into a suspension with saline or other solutions to form a microbubble contrast material. We chose this option to diminish the risk of ischemia caused by a gas bolus replacing blood in the veins. The CO_2_ is approx. 20 times more soluble in blood than oxygen and even more than air. This allows us to use a large amount of gas for vein visualization. The use of foam is gentler than the complete replacement of blood with gas, but requires a greater volume of contrast agent, which is only partially composed of CO_2_. The gas in the form of foam also has a much greater surface/volume ratio, which allows its more rapid dissolution in blood with a lower risk of tissue ischemia.

Air-based gas foam is frequently used for the contrast ultrasound diagnosis of intracardial shunts (PFO, ASD) in cardiology [15,16]. Large volumes of CO_2_ foam are used for the sclerotherapy of plaque-closed veins in patients [17]. There is also one report of a CO_2_ foam self-preparation device for angiography of the lower abdomen [18]. Our work aims to also use CO_2_ microbubble contrast for vein visualization in the supradiaphragmatic organs.

Contrast-enhanced ultrasound is a non-invasive imaging technique that uses an exogenous contrast agent based on micron-size microbubbles that enhance the ultrasound signal. Currently a great deal of effort is devoted to developing stable microbubbles that do not interact with the surrounding tissues. Our CO_2_ foam microbubble contrast also has no harmful effects, and its benefit is its rapid dissolution in the blood, which allows us to use a large volume of CO_2_ foam without the long-term obstruction of veins and tissue ischemization.

The data obtained in NLC Mode provided important parameters such as Peak Enhancement (PE) and Wash-in Rate (WiR) that were assessed for all ratios of CO_2_ foam. The Peak Enhancement (PE) parameter depends on the microbubble size and represents the maximum intensity of the echo power. A higher PE value also indicates higher contrast (blood) flow. Wash-in Rate is related to the perfusion speed, and is dependent on the contrast delivery system (automatic bolus injection or continual contrast inflow).The Wash-In Rate parameter can be interesting if the foam has different-sized bubbles relative to the tubing diameter. The Wash-in Rate also shows how steep the slope is during perfusion. These parameters are also valuable instruments for the identification of sentinel lymph nodes cancer [19]. Similarly, Świtalska et al. describe both above-mentioned parameters for the assessment of tumor perfusion. Higher WiR values are related to more vascularized tissues and indicate higher perfusion speed [20].

The carbon dioxide microbubbles in the foam form dissolved within a few minutes, which was observed in vivo. The reason for the shorter lifetime of the foam is the high pressure and blood velocity in the blood circulatory system. Moreover, part of the CO_2_ can be absorbed in lungs. This claim explains the shorter circulation time in comparison with commercial contrast. We decided to use a 2:1 ratio between CO_2_ and DMEM medium because of the reasonable stability and gas content in the mixture. Under those conditions, the microbubbles were stable for a longer time than is necessary for intravenous administration and imaging. Microbubbles allow us to monitor their venous flow and their presence in heart chambers. When administered into the veins (tail vein, inferior vena cava), the CO_2_ foam contrast was clearly visible in the right atrium, while injection into the infrarenal aorta did not lead to heart contrast. The microbubbles are safely dissolved during passage through the capillary system and did not cause any harmful effects or pain to the laboratory animals. We did not find any histological lesions in the brains of rats after contrast agent injection. The use of CO_2_ foam applied to venous or arterial circulation thus seems to be a safe and effective approach for contrast-enhanced ultrasonic angiography. The limitation of the CO_2_ foam is its shorter lifetime in comparison with commercially available contrasts for US or CT. This contrast is not convenient for the conventional CT imager which has a longer acquisition time in comparison with real-time US imaging.

In addition, CO_2_ microbubbles are employed as a contrast agent for the imaging of soft tissues by phase contrast imaging (PCI). For example, Tang et al. describe preclinical imaging of the portal vein using PCI in rats. They prepared CO_2_ foam through the chemical reaction between citric acid and sodium bicarbonate, and finally the microbubbles were encapsulated by egg white proteins [12].

This preliminary work describes how to easily prepare and use the CO_2_ foam for CEUS imaging. The next step can be replacement of FBS-enriched DMEM medium by peptides or other proteins because the foam prepared without FBS is not stable (breaks down very quickly within a few seconds).

A promising future use of CO_2_ microbubbles can also be as a bimodal contrast agent after adding fluorescent dyes into the medium [21]. With this procedure, the microbubbles can be utilized for multimodal imaging using CEUS, photoacoustic imaging or optical fluorescence imaging. The one prospective clinical application of this imaging approach can be imaging of endovascular aneurysm (EVAR).

## 5. Conclusions

Our approach for the preparation and subsequent administration of an ultrasound contrast agent based on carbon dioxide foam achieves high ultrasound echoes inside the rat heart after injection into the tail vein as well as direct administration of CO*_2_* foam into the cannulated inferior vena cava. No impact of the administered contrast agent upon potential prolonged treatment is another benefit of this CO_2_ contrast approach. In the future, we would implement the described approach of CO_2_ foam preparation for the imaging of, e.g., aortic arch and its branches.

## Figures and Tables

**Table 1:** Non-linear contrast imaging results after tail vein administration.

Contrast–Compartment	Peak Enhancement (a.u.)	Wash-in Rate (a.u.)
Vevo MicroMarker–RA Vevo MicroMarker–LA	636 ± 141 929 ± 264	447 ± 153 2645 ± 2037
Vevo MicroMarker–A Vevo MicroMarker–LV	78 ± 42 351 ± 200	53 ± 37 313 ± 250
2 mL CO_2_ Foam–RA 2 mL CO_2_ Foam–LA 2 mL CO_2_ Foam–A 2 mL CO_2_ Foam–LV	2480 ± 1030 363 ± 230 54 ± 13 179 ± 77	3400 ± 2684 1688 ± 1596 72 ± 42 79 ± 27

Peak Enhancement (a.u.) and Wash-in Rate (a.u.) for Vevo MicroMarker contrast and CO_2_ foam determined inside the right atrium (RA), left atrium (LA), aorta (A) and left ventricle (LV) obtained by analysis in Vevo CQ (*n* = 3).

## Data Availability

Raw data are available upon request.
